# Tuberculosis Exposure among Evacuees at a Shelter after Earthquake, Japan, 2011

**DOI:** 10.3201/eid1905.121137

**Published:** 2013-05

**Authors:** Hajime Kanamori, Noboru Aso, Satoko Tadano, Miyoko Saito, Hiroo Saito, Bine Uchiyama, Noriomi Ishibashi, Shinya Inomata, Shiro Endo, Tetsuji Aoyagi, Masumitsu Hatta, Mitsuhiro Yamada, Yoshiaki Gu, Koichi Tokuda, Hisakazu Yano, Hiroyuki Kunishima, Yoichi Hirakata, Takao Saijyo, Miho Kitagawa, Mitsuo Kaku

**Affiliations:** Miyagi Cardiovascular and Respiratory Center, Kurihara, Japan (H. Kanamori, N. Aso, H. Saito, B. Uchiyama, Y. Hirakata);; Tohoku University Graduate School of Medicine, Sendai, Japan (H. Kanamori, H. Saito, N. Ishibashi, S. Inomata, S. Endo, T. Aoyagi, M. Hatta, M. Yamada, Y. Gu, K. Tokuda, H. Yano, H. Kunishima, M. Kitagawa, M. Kaku);; Miyagi Prefectural Government Kesennuma Public Health Center, Kesennuma, Japan (S. Tadano, M. Saito, T. Saijyo)

**Keywords:** tuberculosis and other mycobacteria, bacteria, TB, disasters, emergency shelter, earthquake, Japan, LTBI, exposure, latent tuberculosis infection, latency, Mycobacterium tuberculosis

## Abstract

Tuberculosis was diagnosed in a person who had stayed in a shelter after the 2011 Great East Japan Earthquake. A contact investigation showed that the prevalence of latent tuberculosis infection among other evacuees at the shelter was 20%. Our report underscores the importance of tuberculosis prevention and control after natural disasters.

In the aftermath of the March 11, 2011, Great East Japan Earthquake and subsequent tsunami, many survivors were forced to live in shelters under harsh and unsanitary conditions. The occurrence and outbreaks of infectious diseases at shelters after the earthquake were of concern because conducting standard precautions was difficult and access to health care was poor. Influenza outbreaks and an increase in pneumonia cases in shelters were reported after the earthquake (*1*,*2*), and infection control activities were required to support shelters in efforts to minimize infectious diseases (*3*). We report a case of active pulmonary tuberculosis (TB) in a person who stayed at a shelter after the 2011 Great East Japan Earthquake and the results of an investigation of the prevalence of latent tuberculosis infection (LTBI) among evacuees and others who were exposed to this patient.

## The Study

The index case-patient was an 87-year-old woman with congestive heart failure who was referred from a disaster medical assistance team and admitted to our hospital on April 6. She reported leg edema starting at the end of February and a cough beginning in early March. On March 11, the day of the earthquake and resulting tsunami, she spent a night in a shrine near her home, which had been completely destroyed. She had also lost all of her daily medications. She stayed at a disaster shelter with family members and other evacuees on March 12–15 (3 days) and then moved to her daughter’s home. She reported that she experienced fever, headache, general malaise, appetite loss, “terrible” cough, sputum, and dyspnea on April 4; on April 6, she visited the disaster medical assistance team.

Chest radiograph and computed tomography scan were performed, and results revealed extensive infiltrative shadows with air bronchograms in the right lung; results were normal for the left lung. Pneumonia was diagnosed, and an empirical therapy of intravenous antimicrobial drugs was initiated, pending further evaluation for pulmonary tuberculosis. Sputum smear testing showed acid-fast bacilli (AFB) graded Gaffky 2 (grade 1+ on the World Health Organization scale) (*4*). *Mycobacterium tuberculosis* complex infection was identified by PCR. The patient was placed in a negative-pressure single room and treated with a 3-drug regimen of isoniazid, rifampin, and ethambutol. Sputum culture yielded *M. tuberculosis* susceptible to the drugs administered. The patient improved and was discharged on June 17 after sputum smears were repeatedly negative for AFB. She continued to receive directly observed treatment at our outpatient clinic and completed 9 months of treatment.

The shelter at which the patient stayed after the earthquake was small (60 m^2^), and ≈50 evacuees stayed there at the time she was there. Ventilation was poor because the weather was cold and windows were not opened. Mask supply was insufficient, and most persons did not wear masks or did not wear them properly. Obtaining information on persons who had contact with the index case-patient was difficult because many evacuees were exhausted from stress or had moved to secondary shelters or relatives’ home by the time we visited the shelter. In cooperation with a manager of the shelter and local government, a contact investigation for the index case-patient patient was conducted during June–August 2011 (2–4 months after the last possible exposure) to identify LTBI; a total of 62 contact persons were found. Three contacts (an infant and children <7 years of age) had a tuberculin skin test (TST), and the remaining 57 contacts (persons >7 years of age) underwent whole-blood interferon-γ release assay (IGRA) testing by using QuantiFERON-TB Gold In-Tube (Cellestis, Chadstone, Victoria, Australia), as described (*5*).

Two of 3 contacts tested by TST were positive for tuberculin purified protein derivative (PPD); 9 of 57 contacts tested by IGRA were positive (Table). A 2-month-old infant, a family member of the index case-patient, had been initially tested in May and was PPD negative but received isoniazid therapy for LTBI because the infant had close and frequent contact with the index case-patient. Two months after the 6 months of treatment was completed (8 months after treatment was initiated), results of a repeat TST on this infant were positive. 

**Table:** Sample results from investigation of TB exposure among evacuees at shelter and relatives who had contact with index case-patient after earthquake, Japan, 2011*

Contact type	Age, y/sex	TB screening†		Intervention
		Type	Result	
Evacuee‡	85/M	NA	NA	Follow-up
Evacuee‡	57/F	IGRA	Negative	Complete
Evacuee‡	32/M	IGRA	Negative	Complete
Family	57/M	IGRA	Negative	Complete
Family	55/F	IGRA	Negative	Complete
Family	30/M	IGRA	Negative	Complete
Family	32/F	IGRA	Negative	Complete
Family	11/F	IGRA	Negative	Complete
Family	4/F	TST	Positive	Follow-up
Family	<1/F	TST	Negative	LTBI treatment
Evacuee	64/M	IGRA	Negative	Complete
Evacuee	61/F	IGRA	Negative	Complete
Evacuee	29/M	IGRA	Negative	Complete
Evacuee	56/F	IGRA	Negative	Complete
Evacuee	86/F	IGRA	Negative	Complete
Evacuee	48/M	IGRA	Negative	Complete
Evacuee	54/F	IGRA	Borderline	Follow-up
Evacuee	63/F	IGRA	Negative	Complete
Evacuee	73/M	IGRA	Borderline	Follow-up
Evacuee	59/F	IGRA	Negative	Complete
Evacuee	60/M	IGRA	Negative	Complete
Evacuee	56/M	IGRA	Negative	Complete
Evacuee	63/M	IGRA	Borderline	Follow-up
Evacuee	60/F	IGRA	Borderline	Follow-up
Evacuee	74/M	IGRA	Positive	Follow-up
Evacuee	67/F	IGRA	Negative	Complete
Evacuee	73/M	IGRA	Borderline	Follow-up
Evacuee	40/M	IGRA	Borderline	Follow-up
Evacuee	38/F	IGRA	Negative	Complete
Patient	81/F	IGRA	Negative	Complete
Evacuee	76/M	IGRA	Positive	Follow-up
Evacuee	70/F	IGRA	Negative	Complete
Evacuee	65/M	IGRA	Negative	Complete
Evacuee	62/F	IGRA	Positive	LTBI treatment
Evacuee	43/M	IGRA	Positive	LTBI treatment
Evacuee	75/F	IGRA	Negative	Complete
Family	28/M	IGRA	Negative	Complete
Evacuee	73/M	IGRA	Negative	Complete
Evacuee	62/F	IGRA	Positive	Follow-up
Evacuee	91/F	IGRA	Positive	LTBI treatment
Evacuee	91/F	IGRA	Positive	LTBI treatment
Evacuee	78/M	IGRA	Positive	LTBI treatment
Evacuee	79/F	IGRA	Negative	Complete
Evacuee	49/M	IGRA	Positive	LTBI treatment
Evacuee	38/M	IGRA	Negative	Complete
Evacuee	70/M	IGRA	Negative	Complete
Evacuee	66/F	IGRA	Borderline	LTBI treatment
Evacuee	42/F	IGRA	Negative	Complete
Evacuee	13/F	IGRA	Negative	Complete
Evacuee	7/M	TST	Positive	Follow-up
Evacuee	74/F	IGRA	Negative	Complete
Patient	96/F	IGRA	Negative	Complete

*A complete list of contact persons is available in the expanded Table online (wwwnc.cdc.gov/EID/article/19/5/12-1137-T1.htm). Contact persons were 50 evacuees (3 family members and 47 others) at the shelter, 8 family members at the index case-patient’s daughter’s home, 2 health care personnel (rescue worker and doctor) who rode with the patient in an ambulance, and 2 patients admitted to the same room as the index case-patient in the hospital. TB, tuberculosis; NA, not available; IGRA, interferon-γ release assay; TST, tuberculin skin test; LTBI, latent tuberculosis infection; border, borderline.  †TST or IGRA results were not available for 2 contacts; 1 refused to receive IGRA, and 1 did not undergo testing because of advanced age. IGRA results were evaluated according to the Japanese guideline for using the QuantiFERON-TB Gold In-Tube (*4*). ‡Family member of index case-patient.

For PPD- or IGRA-positive contacts, medical examinations and chest radiographs were performed, but no results characteristic of pulmonary TB were found. After a physician explained risks and benefits of prophylaxis to the contacts (or parents), 8 contacts (7 evacuees and 1 family member) received prophylactic treatment for LTBI; the other contacts received follow-up chest radiography. One year after the earthquake, no active TB cases had been observed among the contacts.

## Conclusions

We detected LTBI among evacuees who were exposed to a patient with active TB at a shelter after the 2011 Great East Japan Earthquake. Refugees and populations displaced after natural disasters are particularly vulnerable to TB in developing countries because crowded living conditions and poor nutritional status can facilitate the development and the transmission of TB (*6**,**7*). However, as seen in this report and others (*8*), displaced populations in industrialized countries may also be vulnerable to communicable diseases after natural disasters. Persons in shelters who have TB or suspected TB should be transferred to a medical facility as soon as the illness is detected because isolation and respiratory protection for airborne diseases such as TB is very difficult to implement in shelters (*9*). 

In our case, some physicians and nurses who saw the index patient did not consider the diagnosis of TB or could not perform AFB testing, which may have delayed time to TB diagnosis and resulted in TB spread in the shelter. Health care personnel working in disaster relief must suspect and rapidly diagnose TB and then conduct contact investigations in collaboration with local public health departments. Signs and symptoms and clinical characteristics of TB in the elderly may be atypical (*10*), so continuous medical education and training are needed to maintain the competence of health care personnel to prevent, diagnose, and treat TB in the elderly.

In this investigation, TST or IGRA results were positive (indicating LTBI) in 11 (18.3%) of 60 contacts; 10 (20%) of 50 evacuees at the shelter had positive results. A previous study in Japan found that the IGRA-positive rate was 7.1% for those 40–69 years of age in the general population (*11*). Japan is considered a middle-burden TB country and has a bacille Calmette-Guérin vaccination program; for these reasons, IGRA is more useful than TST in LTBI screening and contact tracing.

Adherence rates for LTBI treatment were low (19%) at a TB clinic in New Orleans after Hurricane Katrina (*8*). This finding indicates a challenging environment for TB control activities after a natural disaster and suggests an increased risk for transmission because of migration and overcrowding. Disrupted health care services, poor access to TB control programs, and difficulty in patient management may lead to poor treatment adherence, which could result in the emergence of drug-resistant TB strains. However, all (8/8, 100%) patients with LTBI who initiated treatment in our investigation adhered to and completed the regimen. Our success in postdisaster TB control measures at this shelter can be attributed, in part, to the efforts of public health nurses in providing education and directly observed treatment despite limited resources and poor health care access in the affected area. Medical institutions and public health departments should work to cooperatively and collaboratively assist shelter in implementing TB care and control activities for evacuees after natural disasters.
